# The risk of cryptorchidism among sons of women working in horticulture in Denmark: a cohort study

**DOI:** 10.1186/1476-069X-10-100

**Published:** 2011-11-14

**Authors:** Pernille Gabel, Morten Søndergaard Jensen, Helle Raun Andersen, Jesper Baelum, Ane Marie Thulstrup, Jens Peter Bonde, Gunnar Toft

**Affiliations:** 1 Danish Ramazzini Center, Department of Occupational medicine, Aarhus University Hospital, Aarhus, Denmark; 2 Perinatal Epidemiology Research Unit, Department of Pediatrics, Aarhus University Hospital Skejby, Denmark; 3 Institute of Public Health, Environmental Medicine, University of Southern Denmark, Odense, Denmark; 4 Department of Occupational and Environmental Medicine, Odense University Hospital, Denmark; 5 Department of Occupational and Environmental Medicine, Bispebjerg University Hospital, Copenhagen, Denmark

**Keywords:** Gardener, greenhouse, male genital malformation, occupational hazard, pesticide, reproduction

## Abstract

**Background:**

Androgens are crucial for normal testicular descent. Studies show that some pesticides have estrogenic or antiandrogenic effects, and that female workers exposed to pesticides have increased risk of having a boy with cryptorchidism. The main objective of the present study was to investigate whether pregnant women exposed to pesticides due to their work in horticulture experience excess risk of having sons with cryptorchidism.

**Methods:**

We conducted a cohort study of pregnant women working in horticulture using four cohorts including one cohort established with data from the departments of occupational medicine in Jutland and Funen and three existing mother-child cohorts (n = 1,468). A reference group was established from the entire Danish population of boys born in the period of 1986-2007 (n = 783,817). Nationwide Danish health registers provided information on birth outcome, cryptorchidism diagnosis and orchiopexy. The level of occupational exposure to pesticides was assessed by expert judgment blinded towards outcome status. Risk of cryptorchidism among exposed horticulture workers compared to the background population and to unexposed horticulture workers was assessed by Cox regression models.

**Results:**

Pesticide exposed women employed in horticulture had a hazard ratio (HR) of having cryptorchid sons of 1.39 (95% CI 0.84; 2.31) and a HR of orchiopexy of 1.34 (0.72; 2.49) compared to the background population. Analysis divided into separate cohorts revealed a significantly increased risk of cryptorchidism in cohort 2: HR 2.58 (1.07;6.20) and increased risk of orchiopexy in cohort 4: HR 2.76 (1.03;7.35), but no significant associations in the other cohorts. Compared to unexposed women working in horticulture, pesticide exposed women had a risk of having sons with cryptorchidism of 1.34 (0.30; 5.96) and of orchiopexy of 1.93 (0.24;15.4).

**Conclusions:**

The data are compatible with a slightly increased risk of cryptorchidism in sons of women exposed to pesticides by working in horticulture.

## Background

Cryptorchidism (undescended testis) is the most common genital anomaly in boys [1-3]. The prevalence of cryptorchidism at birth in Denmark differs between samples from 2.4% to 9% depending on definition [4,5] and a prevalence between these numbers are found in other countries [2]. Males who have had uni- or bilateral cryptorchidism have a 2 and 6 fold increased risk of infertility, respectively, and a 4-10 fold increased risk of testicular cancer [6,7].

Cryptorchid testes often descend spontaneously within the first postnatal months due to the surge in gonadotropins and testosterone between the first and the fourth fetal month [3,8]. Any anomaly in the normal descent of the testes may result in cryptorchidism [9,10]. The best documented risk factors are low birth weight, preterm birth, and other congenital malformations [11,12]. A recent study suggests that maternal and environmental risk factors are of major importance [13].

The testes are formed from the undifferentiated immature gonad at around seventh week of gestation [14]. Shortly after the first period of male differentiation the testes starts producing male sex-hormones [insulin-like hormone 3 (INSL3), anti-müllarian hormone (AMH), and androgens] [15]. These hormones play an important role in the normal descent of the testis [2,3,6,15]. For humans it is hypothesized that androgen activity around weeks 8-14 of gestation programs later transinguinal descent during weeks 26-35 [16].

Some pesticides have known estrogenic and/or antiandrogenic effects in experimental studies [17-20]. Studies in rodents demonstrate that exposure to estrogenic or antiandrogenic compounds during gestation may cause malformations of the genitals in male fetuses, including cryptorchidism [21-23]. Among these compounds are several fungicides such as vinclozolin [24], prochloraz [25] and procymidon [21].

Few studies have indicated an increased risk of cryptorchism among sons of mothers potentially exposed to pesticides during pregnancy. These studies have estimated the maternal exposure from geographical areas [26,27], farm residence [28], by measurements of persistent pesticides in breast milk [29] or occupation [30]. This study was prompted by a recent prospective study reporting a three fold increased risk of cryptorchidism among sons of 113 female greenhouse workers [31].

Pregnant women working in horticulture are exposed to pesticides, mainly through the skin, during their work with pesticide-treated plants [32]. The aim of this study is to further investigate whether pregnant women with occupational pesticide exposure have an increased risk of giving birth to cryptorchid sons.

## Methods

### Study population

Women who were occupied in greenhouses during pregnancy and consulted a department of occupational medicine and women who participated in one of three previous established cohorts of pregnant women were included in the study. We searched the cohorts for job titles with words including greenhouse, horticulture and gardener (in Danish) and manually checked all job titles to identify women working in horticulture. For all included women, we gathered information about the pregnancy (first day of last menstruation, expected date of birth, gestational week at consultation/interview, parity, previous pregnancies, treatment for infertility, complications during previous pregnancy, malformations in previous children), lifestyle (smoking habits, alcohol consumption, self reported diabetes, medicine intake, self reported body mass index (BMI)), marital status, weekly working hours, specific tasks at work and exposure to pesticides. The information was retrieved from medical records or questionnaires (different for the 4 groups, see below) and from the Danish health registers.

Cohort 1: Since 1981 all pregnant women in Denmark have been offered counseling regarding occupational exposure to chemicals during pregnancy at the departments of occupational medicine [33]. All pregnant greenhouse workers consulting occupational departments in Funen and Jutland in the period between 1982 and 2007 were included (n = 572).

Cohort 2: The Greenhouse Worker cohort from Funen consists of women, who worked in greenhouses and became pregnant in the period from 1996-2000 and participated in the study described by Andersen et al. [31] (n = 314).

Cohort 3: The Danish National Birth Cohort (DNBC) consists of approximately 100,000 pregnant women enrolled during 1996-2002. The women participated in four computer-assisted telephone interviews during pregnancy and post partum, concerning the pregnancy, their lifestyle and occupation. We included women occupied in greenhouses (n = 309) [34].

Cohort 4: The Aarhus Birth Cohort included all women giving birth at the department of Obstetrics at Aarhus University Hospital. The enrolment is ongoing since 1989, and all women answer a comprehensive questionnaire around gestational week 12 about lifestyle, occupation, and previous pregnancies [35]. Pregnant women occupied in greenhouses enrolled in the Aarhus Birth Cohort by January 1st 2009 were included in this particular study (n = 273).

**Figure 1:**
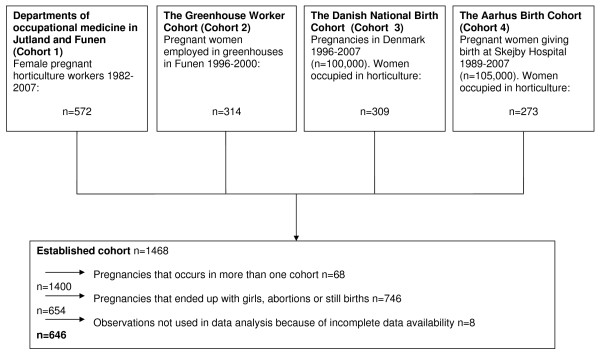
**Flow chart showing the four horticulture worker cohorts included in the study and exclusions**.

From these cohorts we included a total of 1,468 women who worked in horticulture during pregnancy, and who were exposed to various levels of pesticides. Of these, we excluded pregnant women who gave birth to girls, pregnancies ending as an abortion or a stillbirth, pregnant women with missing exposure information and excess observations by women appearing in more than one cohort. After exclusion, we ended up with a cohort of 646 boys and their mothers (Figure 1). We established a large external reference group from the background population including all boys born in Denmark between 1986 and 2007, who were still alive at July 1, 2009 (n = 783,817).

### Exposure classification

Exposure to pesticides (regardless of type of chemical) was assessed by an expert (HRA) blinded to the outcome and divided into three groups (none/low, medium, and high) according to criteria applied in previous studies [31]. Women who had direct contact to pesticides by applying, mixing or dipping cuttings in pesticides were rated as high or medium exposed depending on the exact procedure, the duration of the process, and the use of personal protective equipment. For re-entry activities, the exposure was rated as high if pesticides were used frequently (more than once a week) in the working area and the woman reported often to handle the treated plants without using gloves and to have had work functions with intensive plant contact. Exposure was rated as low if pesticides had not been applied in the working area and the woman had not handled plants that had been treated with pesticides within the last three months. The low exposure group can therefore be considered as an internal non-exposed control group. Exposure situations between these two extremes were rated as medium, e.g. if pesticides were used in the working area but more infrequent (less than once a week) combined with intensive contact to the plants or if pesticides were used frequently but the women had no contact with plant cultures within 24 h after treatment.

In cohort 1, the exposure assessment was based on the available information in the medical journals obtained after consultations at the departments of occupational medicine. The information level varied and did not always allow distinction between high and medium exposure levels. In these cases the women were categorized as medium exposed.

The women in cohort 2 were included in a previous study [31] and were classified into the three categories based on a detailed structured interview of the women at enrolment between gestation week 4 and 10. The information included average weekly working hours, a description of job functions and average weekly time used for each function, personal handling of pesticides (e.g. application and mixing), re-entry intervals before entering greenhouses where pesticides had been applied, procedure for handling of plant cultures recently treated with pesticides, and the use of personal protective equipment. The women were also asked about the type of pesticide products and frequency of use both in the relevant working areas and in the greenhouse as a whole. Supplementary information was collected by telephone contact to the employers.

For cohort 3, the women answered a telephone interview containing the same questions regarding work conditions as used for cohort 2 but without additional information from the employers about the use of pesticides. Since many of the women did not know if and how often pesticides were used, distinctions between high and medium exposure levels were not always possible and as for cohort 1, these women were categorized as medium exposed.

The women in cohort 4 answered a less detailed questionnaire regarding working conditions with a short description of their work task and if they were exposed to pesticides or not. Based on this information, the women were categorized into two categories: pesticide exposed or not exposed.

Most of the women categorized as unexposed worked in the production of tomatoes, cucumbers, or cactuses, where chemical pesticides had been replaced with biological pest control, or in separate greenhouses of other horticultures where pesticides were never or very rarely (once a month or less) used. In addition, some of the women had not been at work for different reasons such as leave or educational purposes.

For all four cohorts, the exposure classification is based on assessment of the total external pesticide exposure. More than 100 different pesticides were used and an assessment of the exposure of the individual compounds taking differences in reproductive toxicity into account was not possible. For all cohorts, the exposure information was collected within the first three months of pregnancy, but exact time periods for exposure during the pregnancy are not known.

### Ascertainment of outcomes and covariates

In Denmark, all residents have a unique personal identifier (the CPR-number), which facilitates linkage between Danish administrative registers and the nationwide health registers operated by the Danish National Board of Health. The Danish Medical Birth Register provided information on date of birth, gestational age, birth weight, and maternal parity. The Danish National Patient Register contains information on all inpatient diagnoses and surgical procedures performed in Denmark since 1977 and similar information on outpatients since 1996 [36]. As one endpoint we used diagnoses of cryptorchidism in ICD-8 (75210) and ICD-10 (Q53, Q531, Q531A, Q532, Q532A, and Q539). Furthermore, as another endpoint we used diagnosed boys who also underwent orchiopexy, as coded by the Danish National Board of Health (55640) and by the Nordic Classification of Surgical Procedures (KKFH00, KKFH01, and KKFH10).

The study was approved by the ethical committee of the Central Denmark Region.

### Statistical analysis

Although cryptorchidism is considered a congenital malformation, not all cases are identified at birth. Some, including recurrences, are diagnosed and treated throughout childhood [37,38]. To account for variation in follow-up periods, we estimated crude and adjusted Hazard Ratios (HRs) by means of Cox regression models, using the boy's age as the time variable. The boys entered the risk set at birth and were followed until their age at first diagnosis, death, emigration from Denmark, or end of follow-up (1 July 2009), whichever came first. We analyzed the effect of occupational pesticide exposure during pregnancy in two ways. First we compared the pesticide exposed horticulture workers to the external control group composed of all Danish boys born in the time period of the study (background population). This allowed us to evaluate if exposed workers had a higher risk than the background population by comparison to a very precise estimate of cryptorchidism and orchiopexy, but without detailed confounder control. We performed two sets of analyses; one which compared the women classified as exposed (exposed from cohort 4 and medium and high exposed from cohort 1, 2, and 3) with the background population, and another which compared two levels of exposure (medium exposure and high exposure) to the background population. A supplementary analysis was performed where members of the control group was drawn as 10 random controls per case matched on calendar year of birth and hospital.

In addition we performed analyses comparing the risk of cryptorchidism and orchiopexy within the combined cohort of horticulture workers, where workers classified as exposed were compared to workers classified as unexposed (unexposed from cohort 4 and low exposed from cohort 1, 2, and 3). This second set of analysis was mainly performed to evaluate if exposed horticulture workers had an increased risk of cryptorchidism and orchiopexy compared to the unexposed horticulture workers. The unexposed workers form an internal control group with similar socioeconomic status and lifestyle factors as the exposed.

The results are presented as crude and adjusted HR with 95% confidence intervals (CI). The following potential confounders were identified a priori and included in all analyses: gestational age at birth (continuous, weeks), calendar year of birth (categorical, 1986-1992; 1993-1999; 2000-2008), maternal age (continuous, years), and cohort of origin (categorical, cohort 1,2,3, and 4). All analyses were done using Stata/SE 10.0 software.

## Results

Characteristics of the study population and the background population are shown in Table 1. Births at the Aarhus and Odense Obstetric departments were overrepresented among horticulture workers compared to the background population, because participants were primarily recruited in hospitals located in these two cities. The calendar years of births are not completely alike either. Most of the boys in the greenhouse cohorts were born between 1993 and 1999. This is because two of the cohorts started in 1996/97. For a part of the population we had information on maternal lifestyle factors as smoking and BMI and relevant medical history, including infertility treatment and diabetes, and found no marked differences in these parameters between exposure groups (table 1). Due to a limited number with this information and the lack of marked differences between exposure groups, these potential confounders were not included in further analyses.

**Table 1:** Characteristics of sons and their mothers, according to maternal occupational pesticide exposure during pregnancy

	Horticulture workers		Background population (n = 783,817)
		
Sons characteristics	Exposed (n = 502)	Not exposed (n = 144)	
Cryptorchidism prevalence [no. (%)]	15 (3.0)	2 (1.4)	16,900 (2.2)

Orchiopexy prevalence [no. (%)]	10 (2.0)	1 (0.69)	11,836 (1.5)

Birth weight g, mean (standard deviation SD)	3533 (587)	3596 (555)	3529 (611)

Birth length cm, mean (range)	52 (38;58)	53 (44;58)	52 (21;65)

Gestational age at birth < 37 weeks, [no. (%)]	34 (6.8)	11 (7.7)	49,227 (6.5)

Gestational age at interview, mean (range) (n = 516)^a^	12 (1;34)	15 (4;31)	∙∙

**Obstetric department:**			

Aarhus [no. (%)]	101 (21)	20 (14)	38,986 (7)

Odense [no. (%)]	232 (48)	47 (33)	22,830 (4)

Other [no. (%)]	154 (32)	77 (53)	494,261 (89)

**Calendar year of birth**			

1986-1994 [no. (%)]	85 (17)	6 (4)	226,105 (29)

1995-2002 [no. (%)]	258 (51)	69 (48)	249,655 (32)

2003-2008 [no. (%)]	159 (32)	69 (48)	308,057 (39)

**Season of birth**			

Spring (March-May) [no. (%)]	125 (25)	37 (26)	200,253 (26)

Summer (June-August) [no. (%)]	131 (26)	39 (27)	207,249 (26)

Autumn (September-November) [no. (%)]	113 (23)	35 (24)	192,957 (25)

Winter (December-February) [no. (%)]	133 (26)	33 (23)	183,358 (23)

**Sub-Cohort**			

1 [no. (%)]	226 (45)	23 (16)	∙∙

2 [no. (%)]	108 (22)	28 (19)	∙∙

3 [no. (%)]	68 (14)	69 (48)	∙∙

4 [no. (%)]	100 (20)	24 (17)	∙∙

**Parental characteristics**			

Parity ≥ 1 ^a ^[no. (%)]	354 (73)	124 (89)	464,587 (78)

Maternal age, mean (range)	27 (17;42)	28 (17;40)	29 (12;54)

Paternal age, mean (range)	30 (19;52)	31 (20;54)	32 (15;78)

Maternal smoking [no. (%)] (n = 584,203) ^a^	104 (22)	34 (24)	134,781 (23)

Maternal BMI, mean (range) (n = 254) ^a^	23 (15;41)	23 (17;38)	∙∙

Maternal diabetes [no. (%)] (n = 414) ^a^	1 (0.34)	0	∙∙

Fertility treatment [no. (%)] (n = 254) ^a^	13 (7)	4 (6)	∙∙

Weeks worked during pregnancy, mean (range) (n = 45) ^a^	18 (0;32)	21 (4;32)	∙∙

Worked full time [no. (%)] (n = 177) ^a^	102 (91)	57 (88)	∙∙

Previous spontaneous abortion/still birth [no. (%)] (n = 421,006) ^a^	40 (15)	2 (5)	121,305 (29)

Previous induced abortion [no. (%)] (n = 360,891) ^a^	38 (14)	8 (21)	72,054 (20)

^a ^n equals the number of observations, in which the data are available for the given variables.

^b ^Number of times the women have given birth (excl. this one)

Boys whose mothers worked in horticulture during pregnancy had a crude prevalence of cryptorchidism of 3.2% for the pesticide exposed group and 2.0% for the unexposed group. The prevalence in the background population was 2.2% (Table 1).

First we compared the exposed women in the cohort to the background population. The HR of cryptorchidism in sons born from pesticide exposed workers compared to the background population was 1.39 (0.84; 2.31), and the HR of orchiopexy was 1.34 (0.72; 2.49). When dividing the occupationally exposed women into two exposure-groups (medium and high) the HR of cryptorchidism was 1.50 (0.81; 2.79) in the medium exposed group and 0.96 (0.24; 3.88) in the highly exposed group compared to the background population (Table 2). Supplementary analysis based on the control sample matched for year of birth and hospital gave essentially same results (data not shown).

**Table 2:** Cryptorchidism in sons of mothers working in horticulture during pregnancy compared to an external control group^a^

Maternal characteristic	Study population, n	Cryptorchidism				Orchiopexy			
		
		n	%	Crude HR	Adjusted HR^b ^(95%CI)	n	%	Crude HR	Adjusted HR^b ^(95%CI)
**Background pop**.	783,817	16,900	2.2	1 (ref.)	1 (ref.)	11,836	1.5	1 (ref.)	1 (ref.)

**Exposed cohort**	502	15	3.0	1.45	1.39 (0.84;2.31)	10	2.0	1.33	1.34 (0.72;2.49)

**Background pop**.	783,817	16,900	2.2	1 (ref.)	1 (ref.)	11,836	1.5	1 (ref.)	1 (ref.)

**Medium exposure**	308	10	3.3	1.62	1.50 (0.81;2.79)	5	1.6	1.11	1.10 (0.46;2.64)

**High exposure**	95	2	2.1	0.93	0.97 (0.24;3.88)	1	1.1	0.65	0.67 (0.09;4.78)

^a ^The control group consisted of all Danish boys born in the study period.

^b ^Results are adjusted for gestational age at birth, calendar year of birth and maternal age.

Then we compared pesticide exposed women to unexposed women working in horticulture (Table 3). The HR of cryptorchidism in the exposed group compared to the unexposed was 1.34 (0.30; 5.96) and the HR of orchiopexy was 1.93 (0.24; 15.4). When exposed women were divided into groups of high or medium exposure, the HR of cryptorchidism in the medium exposed group was 2.18 (0.27; 17.6). HR of cryptorchidism in highly exposed compared to non/low exposed was 1.31 (0.12; 14.6).

**Table 3:** Cryptorchidism in sons of mothers working in horticulture during pregnancy ^a^

Maternal characteristic	Study population, n	Cryptorchidism				Orchiopexy			
		
		n	%	Crude HR	Adjusted HR^b^ (95%CI)	n	%	Crude HR	Adjusted HR^b^ (95%CI)
**Analysis including cohort 4 (n = 646)**									

**Not exposed**	144	2	1.4	1 (ref.)	1 (ref.)	1	0.7	1 (ref.)	1 (ref.)

**Exposed**	502	15	3	1.62	1.34 (0.30;5.96)	10	2	2.31	1.93 (0.24;15.4)

**Analysis excluding cohort 4 (n = 522)**									

**Non/low exposure**	119	1	1	1 (ref.)	1 (ref.)	0	0	∙∙	∙∙

**medium exposure**	308	10	3	2.71	2.18 (0.27;17.6)	5	1	∙∙	∙∙

**high exposure**	95	2	2	1.58	1.31 (0.12;14.6)	1	1.1	∙∙	∙∙

^a ^Internal analysis comparing horticulture workers with different pesticide exposure levels.

^b ^Results are adjusted for gestational age at birth, calendar year of birth, maternal age and the cohort of origin.

The risk of orchiopexy could not be evaluated in relation to three level of exposure among horticulture workers due to no cases in the non/low exposed group (table 3).

An increased risk of cryptorchidism has previously been reported for boys in cohort 2. Therefore we made the analyses stratified for each group individually (table 4), and observed the same trend in cohort 1, 2, and 4. The analyses could not be made for cohort 3, since there were no cases in that cohort. In cohort 2, a statistically significant increased risk of cryptorchidism was observed with a HR or 2.58 (1.07; 6.20). Furthermore the risk of orchiopexy in cohort 4 was also statistically significantly elevated [HR 2.76 (1.03; 7.35)].

**Table 4:** Cryptorchidism in sons of mothers working in horticulture during pregnancy-separate analyses for each cohort^a^

Maternal characteristic	Study population, n	Cryptorchidism				Orchiopexy			
		
		n	%	Crude HR	Adjusted HR^b ^(95%CI)	n	%	Crude HR	Adjusted HR^b ^(95%CI)
**Background pop**.	783,817	16.900	2.2	1 (ref.)	1 (ref.)	11,836	1.5	1 (ref.)	1 (ref.)

**Exposed cohort 1**	226	7	3.1	1.25	1.34 (0.64;2.82)	4	1.8	0.99	1.06 (0.40;2.82)

**Exposed cohort 2**	108	5	4.6	2.83	2.58 (1.07;6.20) ^c^	2	1.9	1.49	1.50 (0.38;6.00)

**Exposed cohort 3**	68	-	-	∙∙	∙∙	-	-	∙∙	∙∙

**Exposed cohort 4**	100	3	3.0	1.44	1.44 (0.46;4.47)	4	4.0	2.72	2.76 (1.03;7.35) ^c^

^a ^Each cohort compared to an external control group of all Danish boys in the study period.

^b ^Results are adjusted for gestational age at birth, calendar year of birth and maternal age.

^c ^HR is statistically significantly different from 1.

## Discussion

Our data indicate that women exposed to pesticides by working in horticulture during pregnancy may at most have a slight to moderate increased risk of giving birth to cryptorchid boys. This was based on the upper limit of the confidence intervals of the most robust estimates, comparing horticulture workers with the background population. Statistically significant elevated risk of cryptorchidism or orchiopexy was observed in two of the four cohorts but not in the combined analysis. The results did not suggest a higher risk in the high exposure group compared to the medium exposed group but this may be explained by low numbers and possible misclassification between the two exposure groups.

The inclusion criteria in the four cohorts differed somewhat. Especially horticulture workers attending occupational health clinics may differ from horticulture workers recruited in the other cohorts. However, in all cohorts the women were recruited before any knowledge on the outcome of their pregnancy making differential selection unlikely. Furthermore, the apparent similar risk estimates in separate cohorts with possible different selection into these cohorts makes it unlikely that the results are driven by selection bias.

Information about the working conditions and pesticide exposure during pregnancy were collected for each mother by interviews or self-administered questionnaires and assessed blinded to the outcomes by experts in occupational exposure assessment. This exposure assessment is probably more accurate than proxies such as job title, but exposure misclassification is still likely. Exposure assessment of horticultural workers is very complex because the exposure varies in time and place depending on e.g., pest attack, season, and type of culture.

Although we have detailed information about working conditions and pesticide use, especially for cohort 2, the average total external pesticide exposure rated for each woman may not reflect the relevant internal exposure. For example, the lack of knowledge about skin penetration and endocrine disrupting properties for most of the pesticides and the exact exposure period during pregnancy hampered the exposure evaluation. Besides, some of the women categorized as highly exposed due to direct contact to pesticides may have lower real exposure due to more awareness and thus a more optimal behavior (e.g., higher frequency of hand washing, correct use of gloves and other protective equipment). These considerations are supported by previously reported results from cohort 2, in which xeno-estrogenic activity in serum from the women was measured as a marker for occupational pesticide exposure [39]. Women categorized as high or medium-level exposed had significantly higher xeno-estrogenic activity in serum than low/unexposed women, but there was no difference between medium and high-level exposed women. For cohort 1 and 3, the exposure information was less detailed and women for whom there were doubts about level of pesticide exposure should be categorized as high or medium-level were categorized as medium-level exposed and some of them are probably misclassified. Hence, while the distinction between the low/unexposed and the exposed (medium or high) groups are considered valid the distinction between medium and high-level exposure groups is more uncertain. Furthermore, it should be acknowledged that some women working in greenhouses might change work functions or left the workplace to reduce exposure after recognition of pregnancy. The exposure categorization was based on the exposure information obtained in the beginning of the pregnancy and for most women the exposure level may be considerably lower later in pregnancy. However, as stated earlier, the suggested male programming window in week 8-14 is suspected to be specifically sensitive to external disturbances, and behavioral changes and protective measures may not have occurred at this early stage in pregnancy.

The cryptorchidism outcome is obtained from the registers of the Danish National Board of Health. The prevalence of cryptorchidism in the registries is likely lower than the true prevalence, indicating under-ascertainment by the register that may be more pronounced for the mild cases. By clinical examination of the boys in cohort 2, seven cryptorchidism cases were detected [31]. Out of these seven boys, one with bilateral non-palpable testicles and two with unilateral suprascrotal or non-palpable testicles at three months of age were identified in the register with a cryptorchidism diagnosis and two of them also with orchiopexy. Four boys with testicles described as high scrotal (one bilateral) at three months, were not registered. This confirms that the registry is not complete and that the less severe cases are not always registered. If this under-ascertainment is independent of the maternal pesticide exposure during pregnancy it will not affect the results obtained in the present study. Balanced under-ascertainment in each exposure group will not lead to biased estimates, but will reduce the power of the study. The diagnosing of cryptorchidism may vary between doctors. This should be taken into consideration in our study, where different doctors in different hospitals have diagnosed the cases. However, we expect a high specificity of cases both diagnosed and operated.

In addition to pesticide exposure and the selected covariates in the present study, several familial and environmental risk factors for cryptorchidism have been identified. In the present study, we assessed maternal smoking and infertility treatment in part of the population and found no marked difference in the prevalence between exposed and unexposed individuals, indicating limited risk of confounding by not taking these factors into account. However, we cannot exclude residual confounding by unmeasured potential risk factors.

The strengths of this study are individual exposure assessment and the use of prospective exposure information. Some previous studies have used a case-control study design which may be useful for identifying a large number of cases and limiting the number of controls when studying a relative rare disease like cryptorchidism. However, if exposure and/or covariate status are ascertained by maternal questioning after the outcome is known, the results of such a study may be biased. To avoid such recall bias, we designed our study as a cohort study with all information about occupational exposure gathered from the mothers during their pregnancy before cryptorchidism could possibly be recognized. Furthermore, the exposure assessment was blinded, so the expert did not know about the outcome, when assessing the exposure.

Andersen et al. [31] reported 3.2 fold higher prevalence (CI 1.4; 7.4) of cryptorchidism in boys of women working in greenhouses in Funen compared to boys born in the Copenhagen area. Our results-based on register-ascertained endpoints-point towards the same conclusion although with lower risk estimates. The greenhouse worker cohort from Funen is included in the present study (cohort 2), and this might contribute to a larger HR than if they had not been included. However, only three of the seven cases from Andersen's study were found in the registry. The HR in cohort 2 was higher than the other cohorts, but the HRs of the other cohorts all pointed towards an increased risk. The significantly increased risk of cryptorchisim observed in cohort 2 and not the other cohorts may be due to more accurate exposure assessment in this cohort. Weidner et al. [30] conducted a case control study, and reported increased risk of cryptorchidism in boys whose mothers were working in gardening. The major strength of that study is the size, but our exposure measurement is more accurate due to the specific assessment of exposure for every woman individually. Garcia-Rodriguez et al. [26] found increased cryptorchidism prevalence in areas of Italy with a high pesticide use. Damgaard et al. [29] conducted a cross sectional case control study and found no differences between cryptorchid and healthy boys in regards to the measured level of persistent organochlorine pesticides in their mother's breast milk. They did, however, report increased levels of the eight most frequent pesticides in breast milk from mothers of cryptorchid boys. Our results are thus rather consistent with previous findings.

## Conclusions

Our study suggests at most a slight to moderate increased risk of having cryptorchid sons if the mother was occupationally exposed to pesticides during pregnancy by working in horticulture. The risk estimates were significantly elevated in two of the subpopulations but not in the combined analysis.

## List of abbreviations

BMI: body mass index (kg/m^2^); DNBC: Danish National Birth Cohort; CI: confidence interval; HR: hazard ratio; SD: standard deviation.

## Competing interests

The authors declare that they have no competing interests.

## Authors' contributions

PG contributed to planning of the study, data collection, statistical analyses, interpretation of results and made the first draft of the manuscript. MSJ contributed to the planning of the study, statistical analysis, interpretation of results and drafting of the manuscript. HRA contributed to the planning of the study, data collection (exposure classification), interpretation of results and drafting of the manuscript. JB contributed to the planning of the study, interpretation of results and drafting of the manuscript. AMT contributed to the planning of the study and interpretation of results. JPB contributed to planning of the study, interpretation of results and drafting of the manuscript. GT contributed to planning of the study, interpretation of results and drafting of the manuscript. All authors critically revised the manuscript and approved the final version.
